# Supplementary material for: Pre-existing cross-reactive immunity to highly pathogenic avian influenza 2.3.4.4b A(H5N1) virus in the United States
Source: Nat Commun. 2025 Dec 8;16:10954. doi: 10.1038/s41467-025-66431-2 (PMC12686434; doi:10.1038/s41467-025-66431-2)
Supplement: Supplementary file 1 — Supplementary Information [file 41467_2025_66431_MOESM1_ESM.pdf]

**Supplementary Material**

# Pre-existing Cross-reactive Immunity to Highly Pathogenic Avian Influenza 2.3.4.4b A(H5N1) Virus in the United States

Zhu-Nan Li[1], Feng Liu[1], Yu-Jin Jung[1], Stacie Jefferson[1], Crystal Holiday[1], F. Liaini Gross[1], Wen-Pin Tzeng[1], Paul Carney[1], Ashley Kates[2], Ian A. York[1], Nasia Safdar[2], James Zhou[3], Marie-jo Medina[3], Vittoria Cioce[3], Christine M. Oshansky[3], C Todd Davis[1], James Stevens[1], Terrence Tumpey[1], Min Z. Levine[1]*

[1] Influenza Division, Centers for Disease Control and Prevention, Atlanta, GA, USA.

[2] Department of Medicine, University of Wisconsin, Madison WI, USA.

[3] Biomedical Advanced Research and Development Authority, Washington, DC, USA

***Corresponding author:** Min Z Levine, PhD. Influenza Division, Centers for Disease Control and Prevention, MS H17-5, 1600 Clifton Road, Atlanta, GA, 30329. Email: mlevine@cdc.gov, Tel: 404-639-3504.

## Supplementary Table S1. Antigens used in the multiplex influenza antibody detection (MIADA) assay

| Assays (N-plex) | Antigen group | Antigen | Virus strain | Type (subtype or lineage) | HA group | Egg or Cell-origin | Ecto/GH HA1 | Source | GISAID/GenBank Accession No. |
|---|---|---|---|---|---|---|---|---|---|
| 12-plex | H1 HA head | H1.WI/2019 GH | A/Wisconsin/588/2019 | A(H1N1)pdm09 | 1 | Cell | GH HA1 | CDC | EPI1661758 |
| 12-plex | | H1.Syd/2021 GH | A/Sydney/5/2021 | A(H1N1)pdm09 | 1 | Cell | GH HA1 | CDC | EPI1957293 |
| 12-plex | H3 HA head | H3.Cam/2020 GH | A/Cambodia/e0826360/2020 | A(H3N2) | 2 | Cell | GH HA1 | CDC | EPI1837753 |
| 12-plex | | H3.Dar/2021 GH | A/Darwin/6/2021 | A(H3N2) | 2 | Cell | GH HA1 | CDC | EPI1857216 |
| 12-plex | H5 HA head | H5.AW/2021 GH | A/American Wigeon/South Carolina/22-000345-001/2021 | A(H5N1) | 1 | Cell | GH HA1 | CDC | EPI1985910 |
| 12-plex | Group 1 HA stalk | Group 1 HA stalk | A/Michigan/45/2015 | A(H1N1)pdm09 | 1 | Cell | HA Stalk | CDC | EPI662594 |
| 12-plex | Group 2 HA stalk | Group 2 HA stalk | A/Singapore/INFIMH-16–0019/2016 | A(H3N2) | 2 | Cell | HA Stalk | CDC | EPI780183 |
| 12-plex | B HA head | BVic WA/2019 GH | B/Washington/02/2019 | B/Victoria lineage | N/A | Clinical | GH HA1 | CDC | EPI1368874 |
| 12-plex | | BViC Aus/2021 GH | B/Austria/1359417/2021 | B/Victoria lineage | N/A | Cell | GH HA1 | CDC | EPI1845793 |
| 12-plex | | BYam Phu/2013 GH | B/Phuket/3073/2013 | B/Yamagata lineage | N/A | Cell | GH HA1 | CDC | EPI529345 |
| 12-plex | NP | A-NP | A/Brisbane/10/2007 | A(H3N2) | N/A | Egg | NP | IRR | EPI176962 |
| 12-plex | Protein A/G | Protein A/G | N/A | N/A | N/A | N/A | N/A | ThermoFisher | N/A |
| 28-plex | H1 HA head | H1.ID/2018 GH | A/Idaho/07/2018 | A(H1N1)pdm09 | 1 | Cell | GH HA1 | CDC | EPI1206974 |
| 28-plex | | H1. WI/2019 GH | A/Wisconsin/588/2019 | A(H1N1)pdm09 | 1 | Cell | GH HA1 | CDC | EPI1661758 |
| 28-plex | | H1.Syd/2021 GH | A/Sydney/5/2021 | A(H1N1)pdm09 | 1 | Cell | GH HA1 | CDC | EPI1957293 |
| 28-plex | | H1.WI/2022 GH | A/Wisconsin/67/2022 | A(H1N1)pdm09 | 1 | Cell | GH HA1 | CDC | EPI2224788 |
| 28-plex | H3 HA head | H3.Cam/2020 GH | A/Cambodia/e0826360/2020 | A(H3N2) | 2 | Cell | GH HA1 | CDC | EPI1837753 |
| 28-plex | | H3.Dar/2021 GH | A/Darwin/6/2021 | A(H3N2) | 2 | Cell | GH HA1 | CDC | EPI1857216 |
| 28-plex | | H3.MA/2022 GH | A/Massachusetts/18/2022 | A(H3N2) | 2 | Cell | GH HA1 | CDC | EPI2096148 |
| 28-plex | H5 HA ectodomain | H5.Indo/2005 Ec | A/Indonesia/05/2005 HA (H5N1) | A(H5N1) | 1 | Egg | Ecto | CDC | EPI376537 |
| 28-plex | | H5.TX/2024 Ec | A/Texas/37/2024 (H5N1) | A(H5N1) | 1 | Egg | Ecto | CDC | EPI3171488 |
| 28-plex | H5 HA head | H5.Indo/2005 GH | A/Indonesia/05/2005 HA (H5N1) | A(H5N1) | 1 | Egg | GH HA1 | CDC | EPI376537 |
| 28-plex | | H5.AW/2021 GH | A/American wigeon/South Carolina/22-000345-001/2021 | A(H5N1) | 1 | Egg | GH HA1 | CDC | EPI1985910 |
| 28-plex | | H5.TX/2024 GH | A/Texas/37/2024 (H5N1) | A(H5N1) | 1 | Egg | GH HA1 | CDC | EPI3171488 |
| 28-plex | B HA head | BVic WA/2019 GH | B/Washington/02/2019 | B/Victoria lineage | N/A | Clinical | GH HA1 | CDC | EPI1368874 |
| 28-plex | | BViC Aus/2021 GH | B/Austria/1359417/2021 | B/Victoria lineage | N/A | Cell | GH HA1 | CDC | EPI1845793 |
| 28-plex | | BYam Phu/2013 GH | B/Phuket/3073/2013 | B/Yamagata lineage | N/A | Cell | GH HA1 | CDC | EPI529345 |
| 28-plex | Seasonal N1 | N1 NA.BR/2018 | A/Brisbane/02/2018 | A(H1N1)pdm09 | N/A | Cell | N1 NA | Sino Biological US Inc | EPI1799927 |
| 28-plex | | N1 NA.WI/2019 | A/Wisconsin/588/2019 | A(H1N1)pdm09 | N/A | Cell | N1 NA | Sino Biological US Inc | EPI1661757 |
| 28-plex | | N1 NA.Syd/2021 | A/Sydney/5/2021 | A(H1N1)pdm09 | N/A | Cell | N1 NA | Sino Biological US Inc | EPI2413554 |
| 28-plex | | N1 NA.WI/2022 | A/Wisconsin/67/2022 | A(H1N1)pdm09 | N/A | Cell | N1 NA | Sino Biological US Inc | EPI2224787 |
| 28-plex | Seasonal N2 | N2 NA.Cam/2020 | A/Cambodia/e0826360/2020 | A(H3N2) | N/A | Cell | N2 NA | Sino Biological US Inc | EPI1837752 |
| 28-plex | | N2 NA.Dar/2021 | A/Darwin/6/2021 | A(H3N2) | N/A | Cell | N2 NA | Sino Biological US Inc | EPI1925254 |
| 28-plex | | N2 NA.MA/2022 | A/Massachusetts/18/2022 | A(H3N2) | N/A | Cell | N2 NA | CDC | EPI2096147 |
| 28-plex | N1 (H5N1) | N1.NA H5.AW/2021 | A/American wigeon/South Carolina/22-000345-001/2021 | A(H5N1) | N/A | Cell | N1 NA | CDC | EPI1985912 |
| 28-plex | | N1 NA H5.TX/2024 | A/Texas/37/2024 (H5N1) | A(H5N1) | N/A | Egg | N1 NA | CDC | EPI3171486 |
| 28-plex | Group 1 HA stalk | Group 1 HA stalk | A/Michigan/45/2015 | A(H1N1)pdm09 | 1 | Cell | HA Stalk | CDC | EPI662594 |
| 28-plex | Group 2 HA stalk | Group 2 HA stalk | A/Singapore/INFIMH-16–0019/2016 | A(H3N2) | 2 | Cell | HA Stalk | CDC | EPI780183 |
| 28-plex | NP | A-NP | A/Brisbane/10/2007 | A(H3N2) | N/A | Egg | NP | IRR | EPI176962 |
| 28-plex | Protein A/G | Protein A/G | N/A | N/A | N/A | N/A | N/A | ThermoFisher | N/A |

GH: HA globular head
Ec: HA ectodomain

**Supplementary Table S2: Sensitivity and Specificity of the MIADA assay in detecting binding antibodies to the HA head of 2.3.4.4b A(H5N1) virus A/American wigeon/SC/22-00345-001/2021**

| MFI Cutoff to 2.3.4.4b AW/2021 H5N1 GH | Sensitivity % ($p^a/n^b$) | Specificity % ($D^c/Y^d$) | J-index[e] |
|---|---|---|---|
| 500 | 100.0 (42/42) | 98.0 (1438/1467) | 0.980 |
| 750 | 100.0 (42/42) | 99.3 (1457/1467) | 0.993 |
| 1000 | 100.0 (42/42) | 99.6 (1461/1467) | 0.996 |
| 1500 | 97.6 (41/42) | 99.9 (1466/1467) | 0.975 |
| 2000 | 93.0 (40/42) | 99.9 (1466/1467) | 0.951 |

[a]Serum samples showed MFIs > cutoff values
[b]42 H5 positive vaccination sera (≥40 neutralizing antibody titers against A/American wigeon/SC/22-00345-001/2021 A(H5N1) 2.3.4.4b wild type virus)
[c]Serum samples showed MFIs <cutoff values
[d]Total 1467 sera were collected from 489 participants in 3 waves from November 2021 to February 2023
[e]: J-index sensitivity+specificity-1

**Supplementary Table S3: Percent amino acid homology of HA1 sequences of A(H5N1) and recent seasonal A(H1N1)pdm09 viruses**

| | A/Texas/37/2024 H5N1 (2.3.4.4b) | A/American wigeon/2021 H5N1 (2.3.4.4b) | A/Indonesia/05/2005 H5N1 (2.1) | A/Wisconsin/67/2022 H1N1 | A/Sydney/5/2021 H1N1 | A/Wisconsin/588/2019 H1N1 | A/Idaho/07/2018 H1N1 |
|---|---|---|---|---|---|---|---|
| A/Texas/37/2024 H5N1 (2.3.4.4b) | N.A | 99 | 88 | 53 | 53 | 53 | 53 |
| A/American wigeon/2021 H5N1 (2.3.4.4b) | 99 | N.A | 88 | 53 | 53 | 53 | 53 |
| A/Indonesia/05/2005 H5N1 (2.1) | 88 | 88 | N.A | 52 | 53 | 53 | 53 |
| A/Wisconsin/67/2022 H1N1 | 53 | 53 | 52 | N.A | 98 | 97 | 95 |
| A/Sydney/5/2021 H1N1 | 53 | 53 | 53 | 98 | *N.A* | 98 | 95 |
| A/Wisconsin/588/2019 H1N1 | 53 | 53 | 53 | 97 | 98 | N.A | 98 |
| A/Idaho/07/2018 H1N1 | 53 | 53 | 53 | 95 | 95 | 98 | N.A |

N/A: non applicable

**Supplementary Table S4: Percent amino acid homology of NA sequences of A(H5N1) and recent seasonal A(H1N1)pdm09 viruses**

| | A/Texas/37/2024 H5N1 (2.3.4.4b) | A/American wigeon/2021 H5N1 (2.3.4.4b) | A/Wisconsin/67/2022 H1N1 | A/Sydney/5/2021 H1N1 | A/Wisconsin/588/2019 H1N1 | A/Brisbane/02/2018 H1N1 |
|---|---|---|---|---|---|---|
| A/Texas/37/2024 H5N1 (2.3.4.4b) | N.A | 99 | 86 | 87 | 87 | 88 |
| A/American wigeon/2021 H5N1 (2.3.4.4b) | 99 | N.A | 86 | 87 | 87 | 88 |
| A/Wisconsin/67/2022 H1N1 | 86 | 86 | N.A | 99 | 99 | 97 |
| A/Sydney/5/2021 H1N1 | 87 | 87 | 99 | N.A | 99 | 98 |
| A/Wisconsin/588/2019 H1N1 | 87 | 87 | 99 | 99 | N.A | 98 |
| A/Brisbane/02/2018 H1N1 | 88 | 88 | 97 | 98 | 98 | N.A |

N/A: non applicable

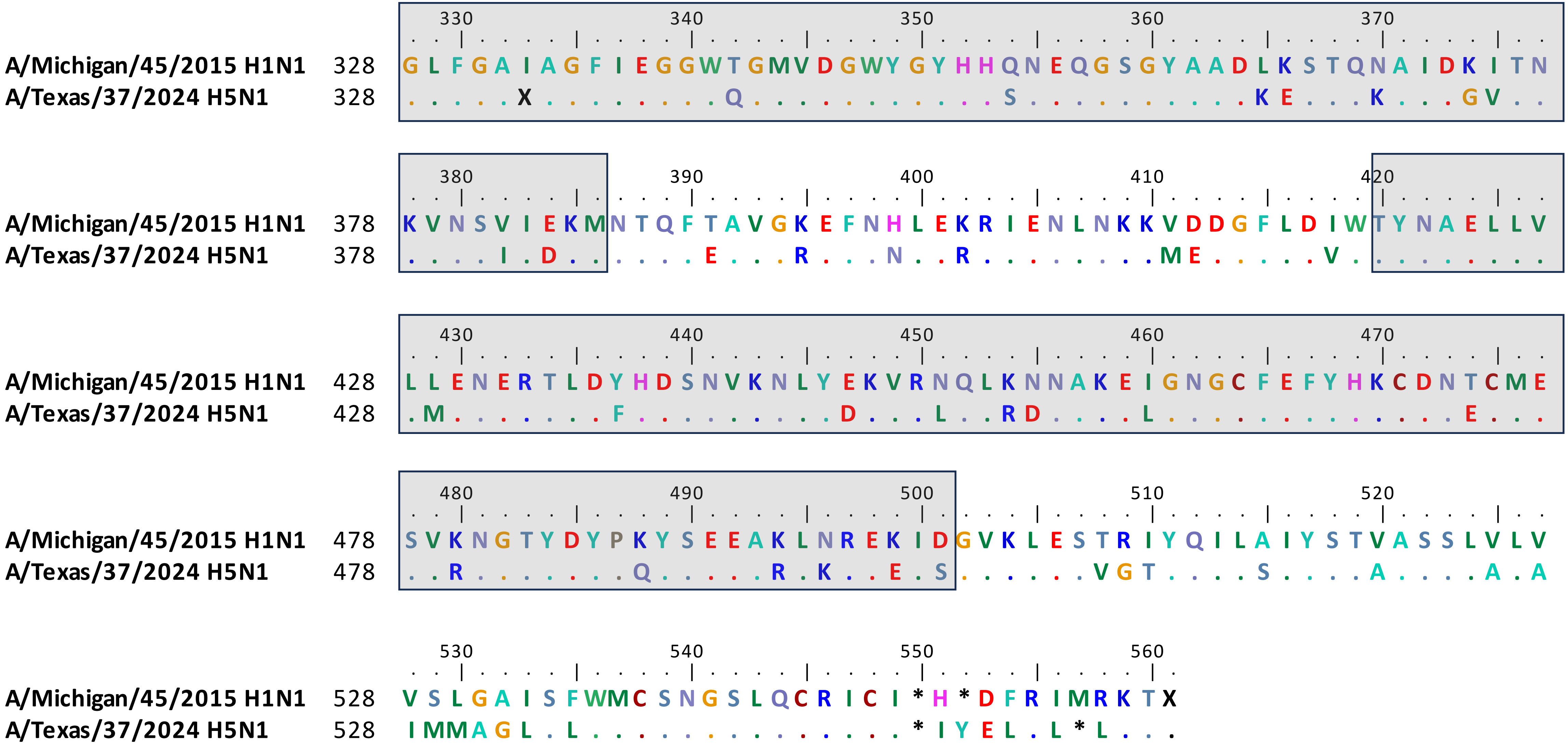


**Supplementary Fig. 1: Amino acid differences between the H1N1 HA stalk and H5N1 HA stalk antigens.** HA stalk sequences from the A(H1N1) and A(H5N1) viruses were analyzed using ClustalW multiple alignment in BioEdit software. HA stalk residuals 328-386 and 420-501 (shown in the grey boxes) were expressed in the HA stalk construct using methods previously described[14]. Identical amino acids referred to the HA stalk sequence of A/Wisconsin/67/2022 H1N1 virus were presented as dots. Amino acid homology of the expressed HA stalk residuals between the two viruses is 84%.

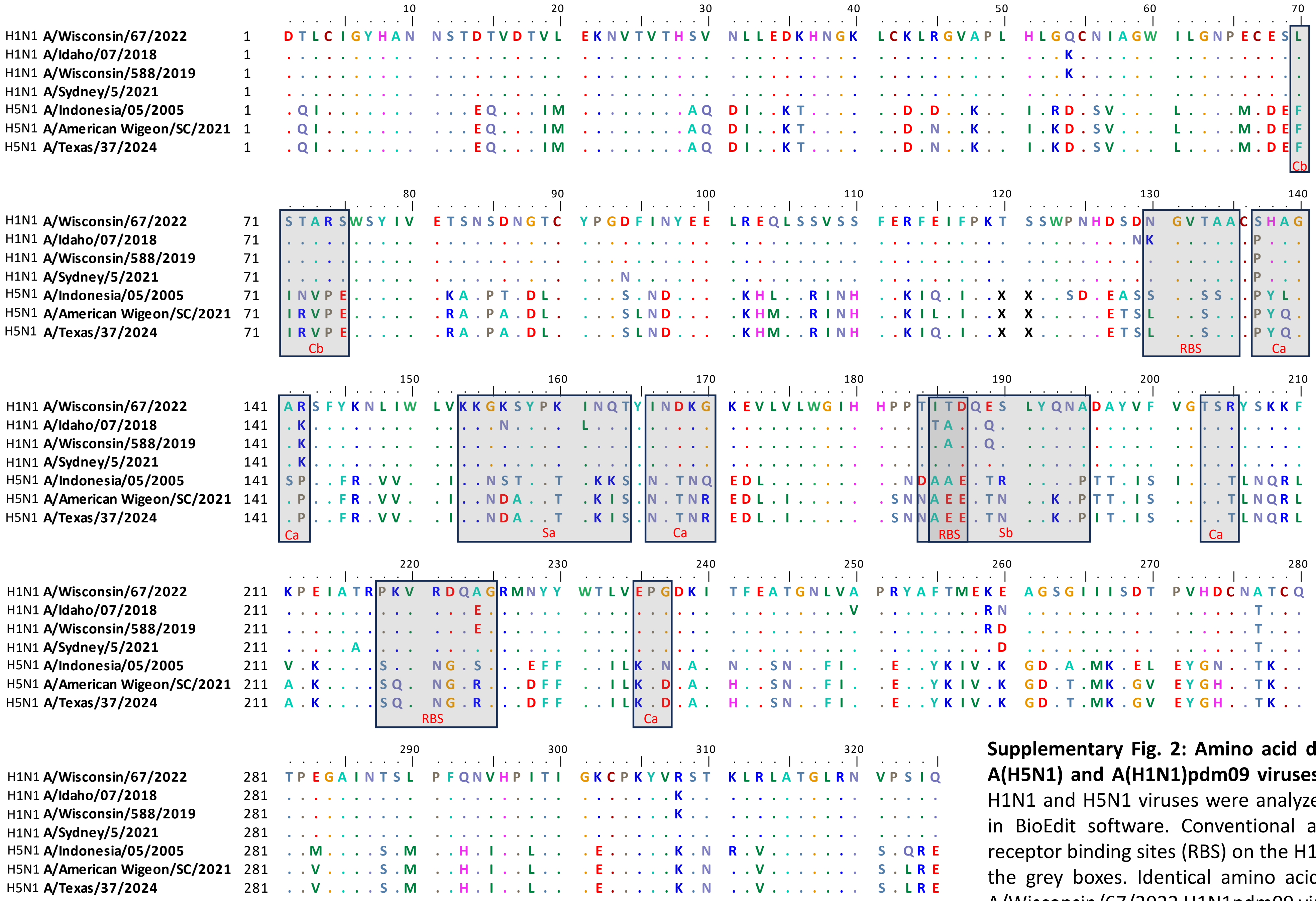


**Supplementary Fig. 2: Amino acid differences between the HA head of A(H5N1) and A(H1N1)pdm09 viruses analyzed.** HA1 sequences from the H1N1 and H5N1 viruses were analyzed using ClustalW multiple alignment in BioEdit software. Conventional antigenic sites (Ca, Cb, Sa, Sb) and receptor binding sites (RBS) on the H1 HA head domain were highlighted in the grey boxes. Identical amino acids referred to the HA1 sequence of A/Wisconsin/67/2022 H1N1pdm09 virus were presented as dots.

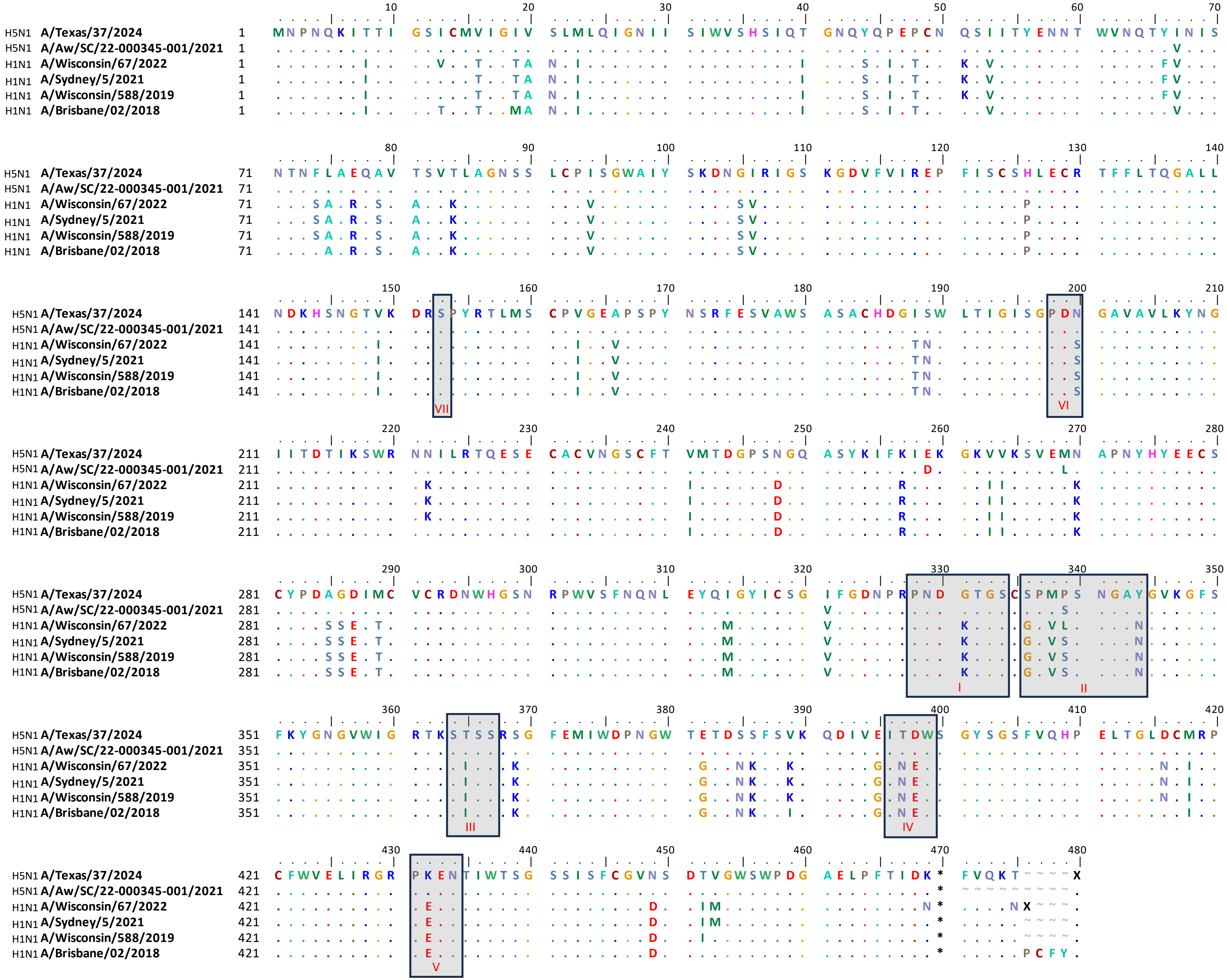


**Supplementary Fig. 3: Amino acid differences among the N1 NA of the A(H5N1) and A(H1N1)pdm09 viruses analyzed.** NA sequences from the H1N1 and H5N1 viruses were analyzed using ClustalW multiple alignment in BioEdit software. Putative NA antigenic sites (I to VII) were highlighted in the grey boxes. Identical amino acids referred to the NA sequence of A/Texas/37/2024 H5N1 virus were presented as dots.

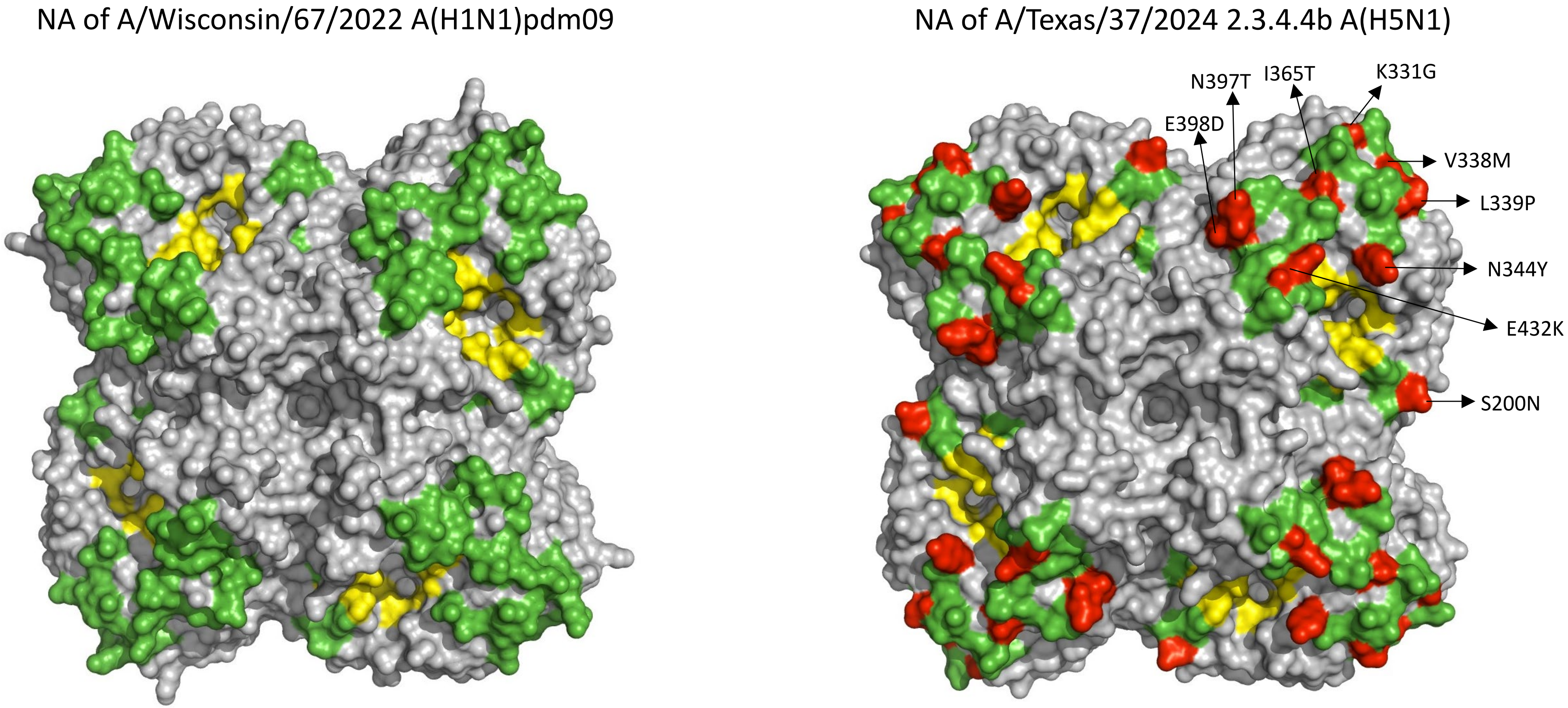


**Supplementary Fig. 4: Comparison of amino acid residues in the potential NA antigenic sites between A/Wisconsin/67/2022 H1N1 pdm09 and A/Texas/37/2024 H5N1 viruses.** The NA tetramers were modeled with PyMOL version 2.1.1 from A/Michigan/45/2015 H1N1 pdm09 (PDB: 7S0I) for A/Wisconsin/67/2022 H1N1 pdm09 NA and from A/American green-winged teal/Washington/195750/2014 H5N1 (PDB: 5HUG) for A/Texas/37/2024 H5N1 NA respectively. Putative NA antigenic sites were shown in green, of which 10 amino acid differences (at site 200, 331, 336, 338, 339, 344, 365, 397, 398, 432) between the two NA sequences were highlighted in red on the modeling of A/Texas/37/2024 H5N1 virus. G336S change was underneath the 338 and 339 sites and was not visible from the top view of this tetramer. The amino acid residues in the enzyme active site were presented in yellow.
